# Coping with the cold: unveiling cryoprotectants, molecular signaling pathways, and strategies for cold stress resilience

**DOI:** 10.3389/fpls.2023.1246093

**Published:** 2023-08-15

**Authors:** Khalil R. Jahed, Amolpreet Kaur Saini, Sherif M. Sherif

**Affiliations:** Alson H. Smith Jr. Agricultural Research and Extension Center, School of Plant and Environmental Sciences, Virginia Tech, Winchester, VA, United States

**Keywords:** cryoprotectants, low temperature stress, ROS, AFPs, transmembrane proteins, antioxidants, ice crystals, cell dehydration

## Abstract

Low temperature stress significantly threatens crop productivity and economic sustainability. Plants counter this by deploying advanced molecular mechanisms to perceive and respond to cold stress. Transmembrane proteins initiate these responses, triggering a series of events involving secondary messengers such as calcium ions (Ca^2+^), reactive oxygen species (ROS), and inositol phosphates. Of these, calcium signaling is paramount, activating downstream phosphorylation cascades and the transcription of cold-responsive genes, including cold-regulated (COR) genes. This review focuses on how plants manage freeze-induced damage through dual strategies: cold tolerance and cold avoidance. Tolerance mechanisms involve acclimatization to decreasing temperatures, fostering gradual accumulation of cold resistance. In contrast, avoidance mechanisms rely on cryoprotectant molecules like potassium ions (K^+^), proline, glycerol, and antifreeze proteins (AFPs). Cryoprotectants modulate intracellular solute concentration, lower the freezing point, inhibit ice formation, and preserve plasma membrane fluidity. Additionally, these molecules demonstrate antioxidant activity, scavenging ROS, preventing protein denaturation, and subsequently mitigating cellular damage. By forming extensive hydrogen bonds with water molecules, cryoprotectants also limit intercellular water movement, minimizing extracellular ice crystal formation, and cell dehydration. The deployment of cryoprotectants is a key adaptive strategy that bolsters plant resilience to cold stress and promotes survival in freezing environments. However, the specific physiological and molecular mechanisms underlying these protective effects remain insufficiently understood. Therefore, this review underscores the need for further research to elucidate these mechanisms and assess their potential impact on crop productivity and sustainability, contributing to the progressive discourse in plant biology and environmental science.

## 1 Introduction

Spring frost is a major environmental stress caused by low temperature combined with dewpoints below freezing points (≤ 0°C), posing substantial economic threat on plants. While the terms “frost damage” and “freeze damage” are commonly used interchangeably, there are slight distinctions between them. Freeze damage is attributed to temperature below freezing points, whereas frost damage is caused by dewpoints below freezing points irrespective of temperature being at or below freezing points (Perry, 1998). However, the damage mechanisms at organ level are comparable in both cases. Consequently, for the purpose of this review, the term “freeze damage” will be employed. At the organ level, the damage occurs when water within plant tissues freezes, forming extracellular ice crystals that result in cell dehydration. Freezing-induced cellular dehydration is the predominant cause of damage in which the cell membranes are disrupted when the dehydration exceeds cell dehydration-tolerance (Pearce, 2001). The severity of damage is determined by multiple factors including plants species, their genetic makeup, dewpoint, surface moisture, probability of an extracellular ice nucleation event, pre-frost environmental conditions, and developmental stage – with flowering being the most susceptible developmental stage (Centinari et al., 2016). Crops can be exposed to two types of frost: radiation and advective – with the former being more common (Liu and Sherif, 2019). Radiation frost is a meteorological phenomenon resulting from the release of thermal energy stored in the soil. This thermal energy is radiated back into the atmosphere during the nocturnal period, characterized by a stable atmosphere and a low wind regime, allowing the formation of a temperature inversion. This atmospheric condition, in conjunction with a reduced dewpoint temperature, leads to a sharp reduction in the air temperature, which falls below the freezing point of water. Advective frost, on the other hand, occurs when a massive system of cold air moves into an area from polar and arctic regions, typically accompanied by wind ≥ 8 km/h. The resultant wind-driven turbulent mixing and cold advection lead to a significant reduction in the air temperature, resulting in the formation of frost.

Crop losses resulting from frost damage represent a significant economic threat to the crop production industry, with frost-related yield reduction being ranked highest among all weather-related disasters in the United States (Centinari et al., 2016; Snyder and de Melo-Abreu, 2005). For example, the one-week Easter freeze in 2007 led to over 2 billion dollars in economic losses due to reduced production of wheat (*Triticum aestivum*) by 19%, peach (*Prunus persica*) by 75%, apple (*Malus domestica*) by 67%, and pecan (*Carya illinoinensis*) by 66% (Liu et al., 2018). Additionally, based on our observations, even a brief frost event persisting only for a few hours can cause substantial damage to various crops. A recent hard freeze event in 2022, lasting 2.5 hours in Virginia, U.S., resulted in an 80% rate of damage to the sweet cherry crop, 15 – 35% damage to apple floral buds, and 15% damage to peach crops (Sherif, 2023). Climate change, leading to increasing global temperatures, exacerbates this threat (Liu et al., 2018), causing alterations in plant phenology such as shortened dormancy and early budburst and flowering (Augspurger, 2013; Ma et al., 2019). Economic losses vary in severity, ranging from partial to complete loss of valuable crops, and can have detrimental impacts on crop quality. Frost-related losses are not limited to specific regions or species; rather, they are a global phenomenon. Notable incidents such as the Easter freeze of 2007 (Liu et al., 2018), the Mother’s Day Freeze (2010), the Killer Frost (2012), and the Polar Vortex (2014) have inflicted billions of dollars in damages on the agricultural industry across North America.

The critical temperature at which crop damage occurs depends on multiple factors, including species specificity, duration of the frost event, and developmental stage (Centinari et al., 2016). For instance, the lower temperature limit at which 90% of floral buds are damaged ranges from -17.6˚C to -3.0˚C for tree fruits like apples, -19.4˚C to -2.8˚C for grapes, -6.1˚C to -0.6˚C for small fruits like strawberries, and -2.8˚C to -0.8˚C for citrus (Snyder and de Melo-Abreu, 2005). Plants at the dormant stage are less susceptible to low temperatures compared to advanced developmental stages. For example, apple floral buds at the silver-tip stage, characterized by slight separation and a shimmery gray color, can tolerate temperatures as low as -17.6˚C. However, at the post-bloom stage, 90% flower mortality can occur at -3˚C. Furthermore, the duration and severity of a frost event have a significant impact on the extent of damage – the more severe and longer it persists, the more damage occurs. Our research, involving a series of experiments investigating frost mitigation strategies, showed that a deleterious frost event in 2023, persisting for 1.5 hours, resulted in approximately 87% damage to apple floral buds, while a 2-hour long frost event in 2021 caused around 65% bud mortality. However, a relatively longer frost event in 2022, lasting around 2.5 hours, resulted in approximately 15 – 35% damage to floral buds. Interestingly, although the duration of frost in 2022 was relatively longer, it coincided with the early developmental stage of the crops, resulting in comparatively lower damage to the crops (Sherif, 2023). These observations suggest that crops at an early developmental stage exhibit increased resilience to extended frost exposure.

Frost protection methods for plants can be categorized into passive and active methods. Passive methods are considered preventive measures applied before the occurrence of frost events (Liu and Sherif, 2019). These methods are more economically efficient compared to active ones and are intended to provide protection over an extended period. Examples of passive methods include site selection, enhancing plant tolerance to frost through the application of cryoprotectant compounds and growth regulators, and the selection of frost-tolerant varieties (cold-hardy and/or late-blooming varieties). On the other hand, active frost protection methods are those applied during or immediately before a frost event with the aim of preventing the temperature from dropping below the freezing point. These methods involve various techniques including heaters (i.e., solid fuel, propane), wind machines, helicopters, surface irrigation, and sprinklers (i.e., over- and under-tree sprinklers). These approaches are designed to actively counteract the effects of frost in real-time.

Specific weather conditions, including wind speed, humidity, and the severity and duration of a frost event, significantly influence the efficacy of both active and passive methods of frost mitigation (Unterberger et al., 2018). For example, wind machines are beneficial for countering radiation frost, but they fail to provide protection against advective frost. Moreover, active methods frequently carry considerable costs, such as those involved in the acquisition and upkeep of necessary equipment. Implementing heaters is one such method, which, while effective, necessitates energy consumption and regular maintenance expenses. Another example is the use of helicopters, the operation and fueling of which can be significantly costly. Certain practical constraints may also emerge, causing some methods to be unsuitable in particular agricultural contexts. An example is the implementation of surface irrigation or sprinkler systems, which might not be feasible in regions with scarce water resources. In addition to these considerations, the environmental impact of these methods, particularly in terms of their energy and water usage, should be evaluated to determine their sustainability. A comprehensive understanding of these limitations is indispensable for making informed decisions about frost protection strategies, as this allows for the consideration of the specific needs and restrictions unique to each agricultural system.

A relatively recent strategy to mitigate frost damage in plants involves the application of chemical compounds, specifically plant growth regulators (PGRs). Over the past half-century, researchers have extensively probed PGRs for their potential in bolstering plant frost tolerance and delaying blooming periods, thereby averting late-spring freezes. Ethephon (2-chloroethylphosphonic acid), for example, is an ethylene-based PGR compound that has shown promising results in delaying bloom and increasing cold hardiness in various fruit and nut trees (Liu and Sherif, 2019; Liu et al., 2021a). Our research involved conducting a set of experiments to investigate the impact of ethephon applications on bloom delay. Our findings revealed that applying ethephon in the fall resulted in a bloom delay of 5-6 days in peaches, varying based on application timing and concentration (Liu et al., 2021a). Such a delay could notably lower the risk of frost damage. Furthermore, our results indicated that ethephon could enhance the cold hardiness of dormant buds, thereby promoting their survival through winter. However, ethephon application does have its drawbacks. For instance, it has been associated with adverse effects like gummosis, terminal dieback, and yield reduction (Liu et al., 2021a). Another frost damage mitigation approach involves applying exogenous cryoprotectant compounds. Yet, the specific physiological and molecular mechanisms that enable these compounds to offer frost protection remain relatively unclear. In this article, we present an in-depth overview of the physiological and molecular mechanisms associated with cryoprotectant compounds. However, gaining a robust understanding of these mechanisms requires a comprehensive grasp of plant cold-sensing mechanisms, natural plant defenses against freezing, and the damaging effects of freezing on plant tissues. Therefore, we also provide a detailed examination of these defense mechanisms and the impact of freezing on plants.

## 2 Cold sensing mechanisms

**Figure 1:**
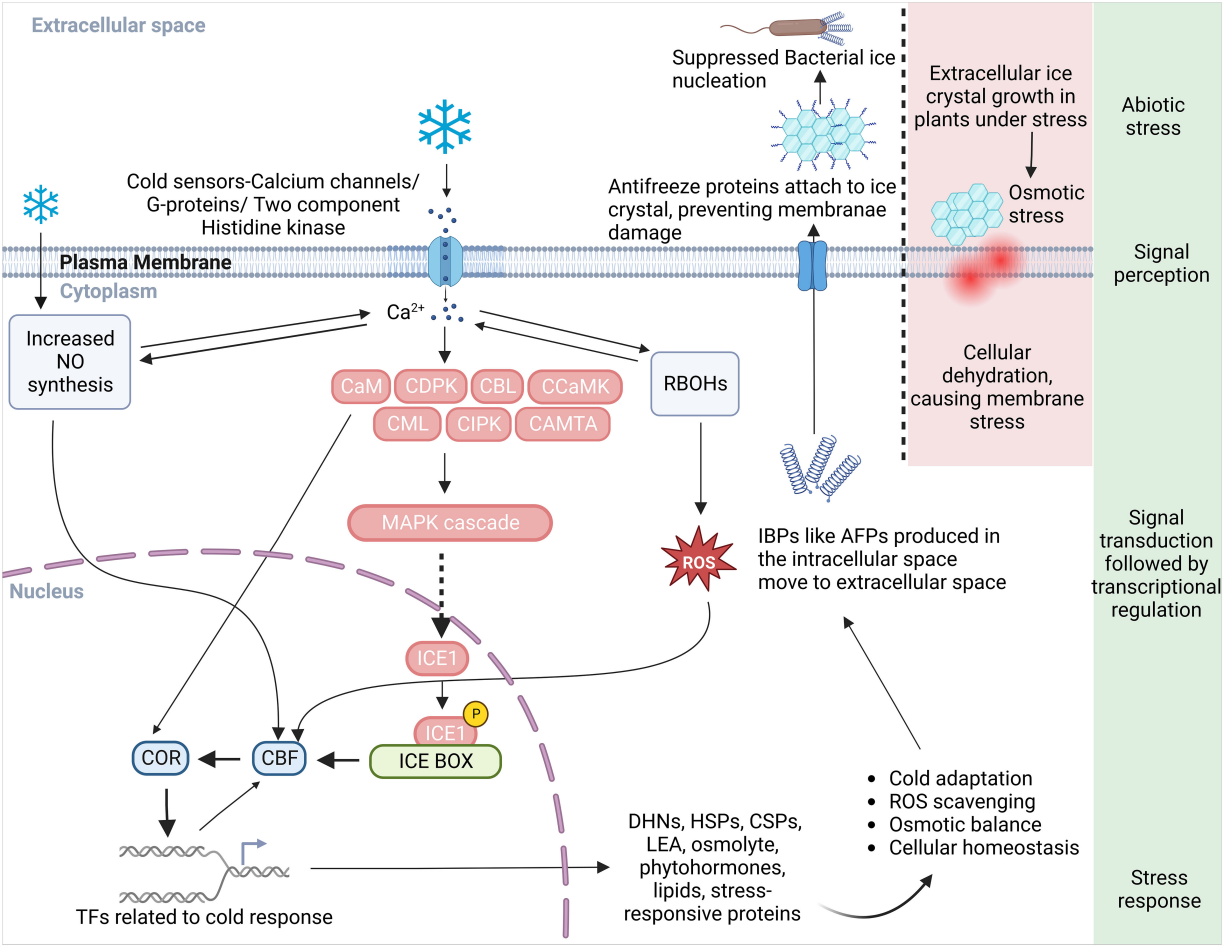
A schematic diagram illustrating an overview of the cold perception and the Ca^2+^ mediated cold responsive signal transduction and response pathways in plant. Plasma membrane receptors and membrane rigidification sense the cold stress and initiate a series of downstream reactions. This cascade activates calcium channels/G-proteins, a two-component histidine kinase, and increases Ca^2+^ influx in the cytoplasm, subsequently stimulating Ca^2+^-related proteins such as CaM, CDPK, CBL, CCaMK, CML, CIPK, CaMTA, and the MAPK signaling pathway. ICE1 interaction with the signaling cascade instigates the ICE-CBF-COR transcriptome machinery’s response. Proteins responsible for the synthesis of cryoprotectants, PKs, phytohormones, and protective proteins like DHNs, AFPs, HSPs, and CSPs, all crucial in cold adaptation, are encoded by the COR genes. For freeze protection in cold-acclimated plants, the process involves IBP induction, secretion of AFPs into the extracellular space, and attachment to ice crystals to inhibit their growth and to prevent freezing linked with bacterial ice nucleation. In non-acclimated plants lacking IBP, large damaging ice crystals form that can rupture the plasma membrane due to cellular dehydration caused by an osmotic gradient that sequesters intracellular water. An increase in Ca^2+^ level due to cold stress also activates RBOHs to produce more ROS and prompts NO synthesis necessary for cold adaptation response. The crosstalk between ROS and Ca^2+^ controls the expression of defense related genes in nucleus. ROS, reactive oxygen species; NO, nitric oxide; RBOH, respiratory burst oxidase homologues; CaM, calmodulin; CDPKs, Ca^2+^ dependent protein kinases; CBLs, calcineurin B-like proteins; CCaMK, Ca^2+^/calmodulin-dependent protein kinase; CML, CaM like; CIPK, CBL-interacting protein kinases; CaMTA, CaM-binding transcription activator; MAPK, mitogen-activated protein kinase; CBFs, C-repeat binding factors; ICE, inducer of CBF expression; COR, cold-responsive; AFP, anti-freeze proteins; IBP, ice-binding proteins; DHN, dehydrin, HSP, heat shock proteins; CSP, cold shock protein; LEA, late embryogenesis abundant protein; TFs, transcription factors.

Plants have evolved intricate mechanisms to identify and respond to abiotic stresses such as cold stress. They perceive shifts in their physical and chemical surroundings like water availability, ion concentration, and temperature, and translate these changes into a biological signal via primary sensory mechanisms (Lamers et al., 2020). Each sensor is typically designed to detect a specific feature of the stress, potentially playing a role in a unique branch of the stress signaling pathway. In this context, the cold-signaling pathway is our focal point for this article. Plants deploy sophisticated multilevel processes in response to a decrease in temperature (Örvar et al., 2000). At the cellular level, cold stress signals are perceived via a variety of receptors that include plasma membrane (PM) rigidification, PM-bound G-protein-associated receptors, and other cold sensors such as Ca^2+^ influx channels, two-component histidine kinases, and changes in protein and nucleic acid conformations or metabolite concentrations (**Figure 1**) (Kazemi-Shahandashti and Maali-Amiri, 2018). These signals give rise to secondary messengers like Ca^2+^, ROS, and inositol phosphates. These messengers subsequently adjust the intracellular Ca^2+^ level. Changes in the cytosolic Ca^2+^ level are detected by calcium-binding proteins, also known as Ca^2+^ sensors. These sensors engage with their target proteins to relay the calcium signal within the cell. These proteins coordinate the transfer of the cold stress signal, trigger protein phosphorylation cascades, and manage the expression of transcription factors and cold-regulated (COR) genes in plants (**Figure 1**, **Table 1**) (Drerup et al., 2013; Zhang et al., 2014; Ma et al., 2015; Zhu, 2016; Guo et al., 2018).

**Table 1:** A comprehensive list of cold acclimatization genes, transcription factors, and proteins, along with the species of identification, their mode of action, and references, is provided.

Gene/Transcription Factor/Protein	Species	Mode of Action	References
Fruit Trees and Shrubs			
*MdCPK1a*	*Malus domestica* (apple)	Overexpression in tobacco increased freezing, salt, and cold tolerance, root length, and antioxidants, while reducing membrane damage and lipid peroxidation	(Dong et al., 2020)
*PpCBF6*	*Prunus persica* (peach)	Transient overexpression prevented sucrose degradation and increased chilling tolerance in peach fruit.	(Cao et al., 2021)
*PpCBF1*		Overexpression in apple increased freezing tolerance in both non-acclimated and acclimated conditions	(Wisniewski et al., 2011)
*PpyCBF1-3*	*Pyrus pyrifolia* (Asian pear)	Overexpression in Arabidopsis enhanced tolerance to low temperature, salt, and drought stresses while reducing ROS production	(Ahmad et al., 2019)
BB-*CBF*	*Vaccinium corymbosum* (Highbush blueberry)	Overexpression in Arabidopsis enhanced freezing tolerance and induced certain COR genes	(Polashock et al., 2010)
*VvCBF2*	*Vitis vinifera* (grape)	Overexpression in Arabidopsis enhanced cold tolerance	(Takuhara et al 2011)
*VvCBF4*			
*VvCBFL*			
*VvCZFPL*			
*PgCBF3*	*Punica granatum* (pomegranate)	Overexpression in Arabidopsis increased cold resilience by raising proline, total soluble sugar levels, and enzymatic activity (CAT, SOD, and peroxidase), while reducing electrolyte leakage, MDA content, and ROS production	(Wang et al., 2022)
PgCBF7			
*PtCBF*	*Poncirus trifoliata* (trifoliate orange)	Exhibited increased accumulation in response to low temperature	(He et al., 2012)
*MaPIP2-7*	*Musa acuminata* L. (banana)	Overexpression enhanced tolerance to multiple abiotic stresses (low temperature, drought, salt) by maintaining osmotic balance, reducing membrane injury, and increasing chlorophyll, proline, soluble sugar, and ABA	(Xu et al., 2020)
*MusaPIP1;2*		Overexpression improved cold resilience by reducing MDA levels, increasing proline and relative water content, and enhancing photosynthetic efficiency	(Sreedharan et al., 2013)
*DlCBF1-3*	*Dimocarpus longan* (longan)	Overexpression in Arabidopsis improved cold tolerance by increasing proline accumulation, reducing ROS content, and upregulating cold-responsive genes in the CBF pathway	(Yang et al., 2020)
Solanum			
*LeCOR413PM2*	*Lycopersicon esculentum* (tomato)	Overexpression prevented membrane damage by increasing antioxidant enzymes, ROS scavenging, reducing PSII photoinhibition, and improving osmotic regulation, while suppressing by RNAi resulted in increased sensitivity of plans to cold	(Zhang et al., 2021)
*LeGPA1*		Overexpression increased cold tolerance by inducing ICE-CBF pathway genes and enhancing SOD, peroxidase, CAT, proline, and total soluble sugar levels, while reducing ROS production and lipid peroxidation	(Guo et al., 2020)
*AtCBF1*		Heterology expression of the Arabidopsis *AtCBF1* in tomato improved plant resilience to low temperature by inducing CAT1 gene expression and reducing H_2_O_2_ levels. It also enhanced tolerance to oxidative damage from methyl viologen	(Hsieh et al., 2002)
*AtCBF1-3*	*Solanum tuberosum* (potato)	Overexpression of Arabidopsis *AtCBF* genes resulted in improved freezing tolerance in potato	(Pino et al., 2007)
Field Crops			
*ZmDREB1A*	*Zea mays* (maize)	Overexpression in Arabidopsis induced COR genes and conferred plant’s tolerance to cold, drought and high salinity stresses	(Qin et al., 2004)
*OsDREB1A*	*Oryza sativa* (rice)	Overexpression in Arabidopsis conferred plant’s tolerance to cold, drought and high salinity stresses	(Dubouzet et al., 2003)
*OsDREB1A/B*		Overexpression in Arabidopsis improved tolerance to drought, high-salt and low-temperature. Also, it increased the contents of osmoprotectants such as free proline and various soluble sugars	(Ito et al., 2006)
*GmDREB1B*	*Glycine max* (soybean)	Overexpression in Arabidopsis resulted in elevated tolerance to abiotic and environmental stresses such as cold, drought, salinity, and heat	(Kidokoro et al., 2015)
*BaAFP-1*	*Hordeum vulgare* (malting barley)	Increased accumulation observed after cold acclimatization	(Ding et al., 2015)
*IbCBF3*	*Ipomoea batatas* (sweet potato)	Overexpression led to enhanced tolerance to cold, drought and oxidative stress, and showed improved photosynthesis efficiency and reduced hydrogen peroxide levels	(Jin et al., 2017)
*BnCBF5, BnCBF 17*	*Brassica napus* (rapeseed)	Overexpression improved freezing tolerance, COR genes mRNA accumulation, photosynthesis-related gene transcript levels accumulation, and chloroplast development resulting in increased photosynthetic efficiency and capacity	(Savitch et al., 2005)
*MfLEA3*	*Medicago falcata* (yellow alfalfa)	*MfLEA3* was induced by cold, dehydration, and ABA. Its constitutive expression enhanced tolerance to cold, drought, and high-light stress in transgenic tobacco plants, along with higher CAT activity.	(Shi et al., 2020)
Ornamental Plants			
*SikCOR413PM1*	*Saussurea involucrata* (snow lotus)	Overexpression in tobacco enhanced cold tolerance through Ca^2+^ signaling and membrane stabilization	(Guo, et al., 2019a)
*SiDHN*		Overexpression in tomato enhanced cold tolerance by preserving cell membrane integrity, increasing chlorophyll a and b contents, carotenoid, reducing chlorophyll photo-oxidation and ROS accumulation, and improving antioxidant enzyme activity and photochemical electron transfer efficiency	(Guo, et al., 2019b)
Herbaceous Plants			
*AtCBF2*	*Arabidopsis thaliana*	Loss-of-function mutants resulted sensitivity to freezing after cold acclimatization and high salinity	(Zhao et al., 2016)
*OST1*		Overexpression enhanced freezing tolerance, while *ost1* mutants showed freezing hypersensitivity. Cold-activated OST1 phosphorylates ICE1 and boosts its stability and transcriptional activity in the CBF pathway.	(Ding et al., 2018)
*Phospholipase Dδ (PLDδ)*		Overexpression increased freezing tolerance, while knockout increased sensitivity. PLDδ gene is involved in membrane lipid hydrolysis, contributing ~20% to phosphatidic acid production; its overexpression enhanced phosphatidic acid production	(Li et al., 2004)
*AtDREB1A*	*Nicotiana tabacum* (tobacco)	Overexpression of Arabidopsis *AtDREB1A* gene enhanced drought and cold stress tolerance in tobacco by inducing abiotic stress-related genes and interacting with the dehydration responsive element	(Kasuga et al., 2004)
*PpCBF3*	*Poa pratensis* (kentucky bluegrass)	Transient overexpression in Arabidopsis increased freezing tolerance by reducing electrolyte leakage, H_2_O_2_ and O_2_•− contents, increasing chlorophyll content and photochemical efficacy, and upregulating cold tolerance genes	(Zhuang et al., 2015)
*BdIRI-7*	*Brachypodium distachyon* (Purple false brome)	Knockdown mutants exhibited reduced freezing survival and impaired ice-crystal growth restriction in plants, while showing increased membrane damage and electrolyte leakage.	(Bredow et al., 2016)
*PsCOR413PM2*	*Phlox subulate* (creeping phlox)	Overexpression in Arabidopsis improved low-temperature tolerance by modulating Ca^2+^ flux and influencing the expression of stress-related COR and CBF genes	(Zhou et al., 2018)
Others			
*PttLHY1, PttLHY2*	*Populus tremula* × *Populus tremuloides* (poplar)	RNAi knockdown compromised freezing tolerance during winter dormancy	(IbÁñez et al., 2010)
*EgCBF3*	*Elaeis guineensis* var. Dura × Pisisfera (oil palm)	Overexpression in tomato improved abiotic stress tolerance under *in vitro* conditions	(Ebrahimi et al., 2016)

Plant cold signaling perception and transduction have been extensively studied in recent years, leading to significant advances in our understanding of this complex process (Zhang et al., 2017; Zhao et al., 2017; Ding et al., 2018; Guo et al., 2018). Plants perceive the environmental stimuli and transduce the signal into downstream biological responses by decreasing membrane fluidity, which in turn affects membrane-associated cellular functions (Hou et al., 2014). Different microdomains with specific lipid compositions, including sphingolipids in the plasma membrane, have been identified for their crucial roles in sensing particular temperature ranges (Fabri et al., 2020). Additionally, multiple cold sensors associated with sending temperature changes and cold signaling, such as putative calcium channels, PM-bound G-protein-associated receptors, and plasma membrane-localized receptor-like kinases (RLKs), have been identified in plants (**Figure 1**). Calcium ions enter the cell primarily through transmembrane proteins complex, known as calcium channels, which are crucial sensors for abiotic stress (Görlach et al., 2015). The rigidification of the membrane due to cold stress activates mechanosensitive or ligand-activated calcium channels, leading to a transient Ca^2+^ influx into the cytosol (Wei et al., 2021).

After perceiving cold stress, plants initiate a series of intricate signal transduction events within the cytosol and nucleus. These events rely on secondary messengers like Ca^2+^, ROS, and nitric oxide (NO) to facilitate intracellular signaling and cell-to-cell communication (Marcec et al., 2019). Cold stress-induced Ca^2+^ signatures are deciphered through various pathways involving specific groups of Ca^2+^ sensors such as CaM (calmodulin) and CMLs (CaM-like), CDPKs (Ca^2+^-dependent protein kinases), CCaMK (Ca^2+^-and Ca^2+^/CaM-dependent protein kinase), CAMTA (CaM-binding transcription activator), CBLs (calcineurin B-like proteins) and CIPKs (CBL-interacting protein kinases) (Kudla et al., 2018). The Calcium/CaM-Regulated Receptor-Like Kinase 1, CRLK1, actively modulates MAPK kinase activity and exerts regulatory control over C-repeat Binding Factors (CBFs) regulons, as well as freezing tolerance (Yang et al., 2010). Subsequent downstream reactions in plants are triggered by cold stress encompass the ICE-CBF/DREB1 pathway, the intricate regulation of cold-responsive genes (Miura and Furumoto, 2013), and post-transcriptional and post-translational modifications (Vyse et al., 2020).

In addition to the plasma membrane, chloroplasts may also serve as important sensors for ambient temperature. When temperatures drop, the equilibrium between the ability to capture light energy and the ability to disperse this energy through metabolic processes is disturbed. This disruption results in enhanced excitation pressure on photosystem II (PSII) and subsequently hinders the photosynthetic capacity by inhibiting it (Ivanov et al., 2012). This phenomenon, known as photoinhibition, results in redox imbalances in photosynthetic electron transport and photosynthetic carbon reduction cycles. Eventually, the photosynthetic apparatus is destroyed, and ROS are generated, serving as a secondary messenger (Bhattacharjee, 2019). Disruption of the photosynthesis process under cold-stress conditions significantly reduces crop yield through various mechanisms. The maximum attainable yield, which represents the optimal conversion of captured light energy into biomass, known as photosynthetic efficiency, is compromised under cold stress (Simkin et al., 2019). This reduction in photosynthetic efficiency is primarily attributed to the impairment of key enzymes involved in the carbon fixation cycle, namely fructose-1,6-bisphosphatase (FBPase) and sedoheptulose-1,7-bisphosphatase (SBPase) (Sassenrath et al.,1990; Kingston-Smith et al., 1997; Hutchison et al., 2000). The decreased activity of ribulose-1,5-bisphosphate carboxylase/oxygenase (Rubisco), the primary enzyme involved in carbon fixation, and the reduction in bisphosphate (RuBP) levels are processes observed at low temperatures. Multiple causes contribute to the diminished RuBP regeneration, such as limitations in linear electron transport, chronic photoinactivation, deactivation of stromal bisphosphatases, and end-product limitation. In the case of end-product limitation, the limited synthesis of sucrose, starch, and amino acids leads to the accumulation of phosphorylated intermediates, depletion of the inorganic phosphate pool, and inhibition of ATP synthesis (Leegood and Furbank, 1986; Sharkey et al., 1986; Lemoine et al., 2013). Furthermore, imbalances in the source-sink relationship, where the plant’s source activity surpasses the sink demand, result in decreased mRNA levels, degradation of photosynthetic proteins, and ultimately reduced productivity (Adams et al., 2013; Kurepin et al., 2015). These combined effects contribute to lower crop yields under cold stress conditions.

## 3 Freeze-induced damage in plants: mechanisms and consequences

At freezing temperatures, the aggregation of water molecules within plant cells leads to the formation of stable ice nuclei, which is a critical step in initiating the freezing process. Ice nucleation occurs when these small ice nuclei form a group of membrane proteins known as ice-nucleating proteins (INPs), which act as nucleation sites. These sites promote the formation of extracellular ice crystals by facilitating the proper arrangement of water molecules. In the absence of ice-binding proteins (IBPs), large ice crystals form in the apoplast, which can physically damage plasma membranes. The formation of ice crystals and the subsequent sequestration of intracellular water create an osmotic gradient, leading to cellular dehydration. This loss of cell volume can result in cell collapse or rupture (Pearce, 2001; Larcher, 2003; Snyder and de Melo-Abreu 2005; Zhang et al., 2019). Freeze damage also occurs due to alterations in membrane fluidity caused by the transition of lipid components from liquid into a gel state. This transition reduces membrane selectivity, increases permeability, destabilizes metabolic activities, and inhibits photosynthesis (Larcher, 2003; Zhang et al., 2019).

The magnitude of damage depends on the intensity and duration of freezing conditions and the developmental stage of the plant (Larcher, 2003; Centinari et al., 2016). In some cases, plants can delay the formation of extracellular ice crystals through a process called supercooling, where water in plant tissues remain in a liquid state below the freezing point of water. Supercooling in plants is a sophisticated process, involving various mechanisms working synergistically. It prevents the transition of intercellular water from a liquid to a solid state, effectively suppressing water nucleation and thereby avoiding ice crystallization (Wisniewski et al., 2004; Londo and Kovaleski, 2019). Supercooling is a common phenomenon in woody plants, manifesting in both leaves and the living cells of the xylem, including the xylem ray parenchyma cells. This phenomenon readily takes place in small volumes of water, where the free energy of water is influenced by surface properties, especially in the absence of nucleation particles or agents responsible for initiating ice-crystal formation. This process allows cells to maintain their function, albeit at a relatively reduced rate, and become more tolerant to freeze damage (Cavender-Bares, 2005). However, even in supercooled parts of the plant, progressive dehydration can still occur, indicating that the avoidance of freezing-induced dehydration through deep supercooling is only partial (Pearce, 2001; Larcher, 2003).

Another equally important factor that influences the extent of damage caused by dehydration is the ratio of bound water to free water within plant cells. As freezing-induced dehydration progresses, it can disrupt cell structures associated with the bound water compartment, which is involved in the structural organization of membranes, organelles, and proteins. This disruption can result in irreversible injury to the cells (Elliott et al., 2017). Negative osmotic pressure induces a net movement of water towards the extracellular space, reducing cell volume. In non-acclimated cells, this reduction in cell volume leads to invagination of the plasma membrane and formation of endocytic vesicles, resulting in loss of surface area of the plasma membrane. Upon rewarming, melted water from the extracellular space re-enters the cell, causing the cell to burst before it can regain its original volume (Ambroise et al., 2020).

The antioxidant system in plants also scavenges the excessive amount of ROS, including superoxide (O_2_ **^•^** ^−^), hydroxyl radicals (OH**^•^**), hydrogen peroxide (H_2_O_2_) and singlet oxygen (^1^O_2_) in response to cold stress. The antioxidant compounds play a critical role in maintaining redox homeostasis during cold stress, as optimal levels of ROS are necessary for normal progression of fundamental biological processes including cellular proliferation and differentiation (Tsukagoshi et al., 2010; Mittler, 2017). Under normal conditions, excessive ROS is neutralized through various enzymatic and non-enzymatic antioxidative defense mechanisms. However, when plants are exposed to cold stress, the equilibrium between ROS production and scavenging is disrupted, leading to mitochondrial membrane rigidification (Saha et al., 2015), loss of complex IV (Prasad et al., 1994), DNA damage, and impaired regulation of physiological cell death (Xie et al., 2014; Berni et al., 2019). These disruptive events can cause severe damage to plant cells and their functions, which may affect their survival and reproduction.

## 4 Plant defense mechanisms against freezing: insights and perspectives

Plants possess avoidance and tolerance strategies to mitigate the detrimental effects of freezing temperatures and prevent damage to their cellular structures (Larcher, 2003; Hoermiller et al., 2018). Avoidance mechanisms rely on a variety of cryoprotectant molecules to reduce the intracellular freezing temperature through a plant-specific process called supercooling (Francko et al., 2011). These molecules help maintain the intracellular liquids at sub-zero temperatures supercooled and delay or inhibit the formation of extracellular ice crystals (Larcher, 2003; Wisniewski et al., 2018). Certain plant species have developed sophisticated mechanisms to mitigate freezing damage by utilizing IBPs as a part of their survival strategy. IBPs, also known as antifreeze proteins (AFPs) or ice recrystallization inhibition (IRI) proteins, are a group of low temperature-associated proteins found in various cold-adapted organisms, including plants such as *Lolium perenne, Loliurn perenne and Ammopiptanthus nanus* (Yu et al., 2021), animals, and microorganisms. Plant IBPs possess unique structural features and functional properties that enable them to interact with ice crystals, thereby impeding their growth and inhibiting the movement of water molecules from the intracellular to extracellular space. This binding ability helps prevent cell dehydration and maintains cellular integrity during freezing conditions. Additionally, IBPs exhibit the ability to suppress ice recrystallization, a process that can lead to tissue damage and disrupt plasma membranes. By effectively limiting ice crystal growth and inhibiting ice recrystallization, IBPs play a crucial role in safeguarding plant tissues against freezing temperatures (Bredow and Walker, 2017; Wisniewski et al., 2020). Numerous AFPs have been identified in various plant species, highlighting the diversity of these ice-binding proteins. For example, fpAFP has been discovered in *Festuca pratensis* (Muthukumaran et al., 2011), LpAFP and LpIRI2/3 in *Lolium perenne* (Bredow et al., 2017), BdIRI1-7 in *Brachypodium distachyon* (Bredow et al., 2016), daAFP in *Daucus carota* (Cid et al., 2018) and rsAFP in *Raphanus sativus* (Wisniewski et al., 2020). These specific IBPs exemplify the adaptations of plants to withstand freezing stress and highlight the intricate molecular strategies employed by these organisms to protect themselves from the detrimental effects of low temperatures.

Cold tolerance mechanisms involve acquiring tolerance to low, non-freezing temperatures through a process known as cold acclimation. It is a complex physiological process where plants gradually adapt to decreasing temperatures, resulting in enhanced hardiness and adaptive responses that enable them to withstand freezing conditions. Cold acclimation encompasses coordinated molecular, physiological, and biochemical changes that enhance plant tolerance to cold stress, crucial for survival under low temperature conditions. These adaptations include diverse morphological modifications such as reduced plant height, decreased leaf number, and increased epidermal thickness. Biochemical alterations involve the accumulation of sugars, amino acids, and secondary metabolites. Physiological adjustments encompass decreased photosynthesis, reduced water use efficiency, and altered pigment synthesis. Collectively, these mechanisms enhance a plant’s ability to thrive in cold environments (Fürtauer et al., 2019; Wang et al., 2020; Fang et al., 2021; Guo et al., 2021; Liu et al., 2021b; Satyakam et al., 2022). The regulation of gene expression during cold stress response is a complex network triggered by multiple factors including Ca^2+^ and plant hormones, particularly brassinosteroids (BRs), gibberellins, abscisic acid, jasmonic acid, and ethylene (Arias et al., 2015; Eremina et al., 2016; Hu et al., 2017; Li et al., 2017:1; Juurakko et al., 2021; Mishra et al., 2022; Sarkar and Sadhukhan, 2022). Additionally, plants synthesize secondary metabolites in response to cold stress, such as proline, betaine, and putrescine. These osmolytes act as protective compounds, helping to mitigate the effects of cold stress on plant cells (Lee et al., 2019). Plants also accumulate low-molecular-weight compatible solutes or osmolyte cryoprotectants, such as sucrose, glucose, raffinose, fructose and trehalose. These molecules assist in maintaining cellular osmotic balance and protect cellular structures from cold-induced damage (Karabudak et al., 2014; Lunn et al., 2014; Judy and Kishore, 2016; Roychoudhury and Banerjee, 2016; Suo et al., 2017; Siddique et al., 2018; Liu et al., 2019; Chen et al., 2022). In addition, plants respond to cold stress by increasing the accumulation of suberin and lignin, which contribute to the reinforcement of cell walls and the protection of plant tissues against freezing injury. These modifications are essential for reducing the adverse effects of cold stress on plant growth and development (Trache et al., 2017; Jian et al., 2020; Sun et al., 2021).

The process of cold acclimation in plants also involves plasma membrane rigidification and rearrangement of the cytoskeleton (Knight and Knight, 2012; Ambroise et al., 2020; Satyakam et al., 2022). These alterations trigger increased metabolic activities, initiating signaling pathways that regulate the expression of COR genes (Iqbal et al., 2022; Satyakam et al., 2022). The induction of COR genes is mediated by transcriptional activators known as C-repeat Binding Factors (CBFs), which are controlled by the regulatory module involving the Inducer of CBF Expression (ICE). This regulatory module, referred to as ICE-CBF-COR, represents a central pathway responsible for initiating the cold response in plants (Chinnusamy et al., 2003; Jin et al., 2018). Upon activation, COR genes regulate the synthesis of various protective molecules, including compatible solutes such as soluble sugars and proline, pigments like xanthophylls and carotenoids, amino acids, and cold-responsive proteins such as AFPs, late embryogenesis abundant (LEA) proteins, heat shock proteins (HSPs), cold shock proteins (CSPs), as well as antioxidants and stabilizing proteins like chaperones and dehydrins (DHNs) (Rinehart et al., 2007; Latowski et al., 2011; Yu et al., 2017; Meena et al., 2019; Ambroise et al., 2020). The synthesis and accumulation of these molecules collectively contribute to the development of cold tolerance in plants.

## 5 Cryoprotectants: mechanisms of action and insights in frost mitigation

Over the past two decades, agrochemical companies have developed multiple cryoprotectants (such as KDL, Glacier, diKap, Anti-Stress 550, ThermoMax, FrostGard, etc.) for frost protection – a comprehensive list of the artificial cryoprotectants has recently been summarized in a review by (Román‐Figueroa et al., 2021). These compounds, when externally applied to plants, enhance plant’s freezing avoidance ability through various methods. They create a physical barrier around plant tissues, increase the concentration of endogenous cryoprotectants like metabolites and proteins, and boost the intercellular solute concentration. This elevated solute concentration leads to a depression in freezing point, preventing the formation of extracellular ice crystals and reducing cell dehydration-induced damage caused by apoplastic freezing. It has also been suggested that solute accumulation stabilizes cell membranes and macromolecules, either through direct interaction with the membrane surface or through strong interplay with the surrounding water. The precise mechanism by which these molecules prevent ice crystal formation and cell dehydration is intricate and varies depending on the specific type of cryoprotectant. In this summary, we outline some general mechanisms through which cryoprotectants alleviate freezing damage to plant tissues.

### 5.1 Physical barrier: plant tissue protection using cellulose nanocrystals

Cellulose nanocrystals (CNCs) are nanoscale biomaterials with unique structural characteristics and impressive physicochemical properties. These properties include biocompatibility, biodegradability, renewability, low density, versatile surface chemistry, optical transparency, and enhanced mechanical properties (Trache et al., 2017). CNCs serve as the foundational polymeric motifs of macroscopic cellulosic fibers produced through the acid hydrolysis of cellulose materials. CNCs exhibit a needle-like or rod-like shape and have dimensions on the nanometer scale, typically ranging from a few to several hundred nanometers in length and a few nanometers in width. These materials can be obtained from various sources such as higher plants, marine animals (such as tunicates), and, to a lesser extent, algae, fungi, bacteria, invertebrates, and even amoeba like *Dictyostelium discoideum* (Habibi et a., 2010; Grishkewich et al., 2017). Due to their exceptional attributes, CNCs find applications in diverse fields, including automotive, medicine, construction, marine, aerospace, barrier materials, flexible displays, antimicrobial coatings, biomedical implants, transparent films, pharmaceuticals, drug delivery, electronic component templates, fibers and textiles, separation membranes, supercapacitors, batteries, and electroactive polymers (Trache et al., 2017).

Recently, researchers have explored the use of CNCs as a cryoprotectant for grape and sweet cherry reproductive buds (Alhamid et al., 2018). The application of sprayable CNCs forms a thermal insulation coating around the buds and flowers, resulting in enhanced cold hardness and effective mitigation of frost damage. The protective properties of CNCs can be attributed to their low thermal conductivity (0.061 W m^-1^ K^-1^), which surpasses that of other commonly used frost protection materials. In this study, CNC treatment significantly reduced the damage temperature of grape and sweet cherry buds and improved their resistance to frost damage by 2 – 4°C. Moreover, the application of CNCs delayed the formation of ice nucleation in the buds when exposed to freezing temperatures. The freezing temperature at which ice nucleation occurred was lowered by approximately 3°C in CNC-treated buds compared to untreated buds (Alhamid and Mo 2021). These findings highlight the extensive potential of CNCs as cryoprotectants in enhancing plants’ protective mechanisms and their capacity for freeze protection.

### 5.2 Reducing freezing point

**Figure 2:**
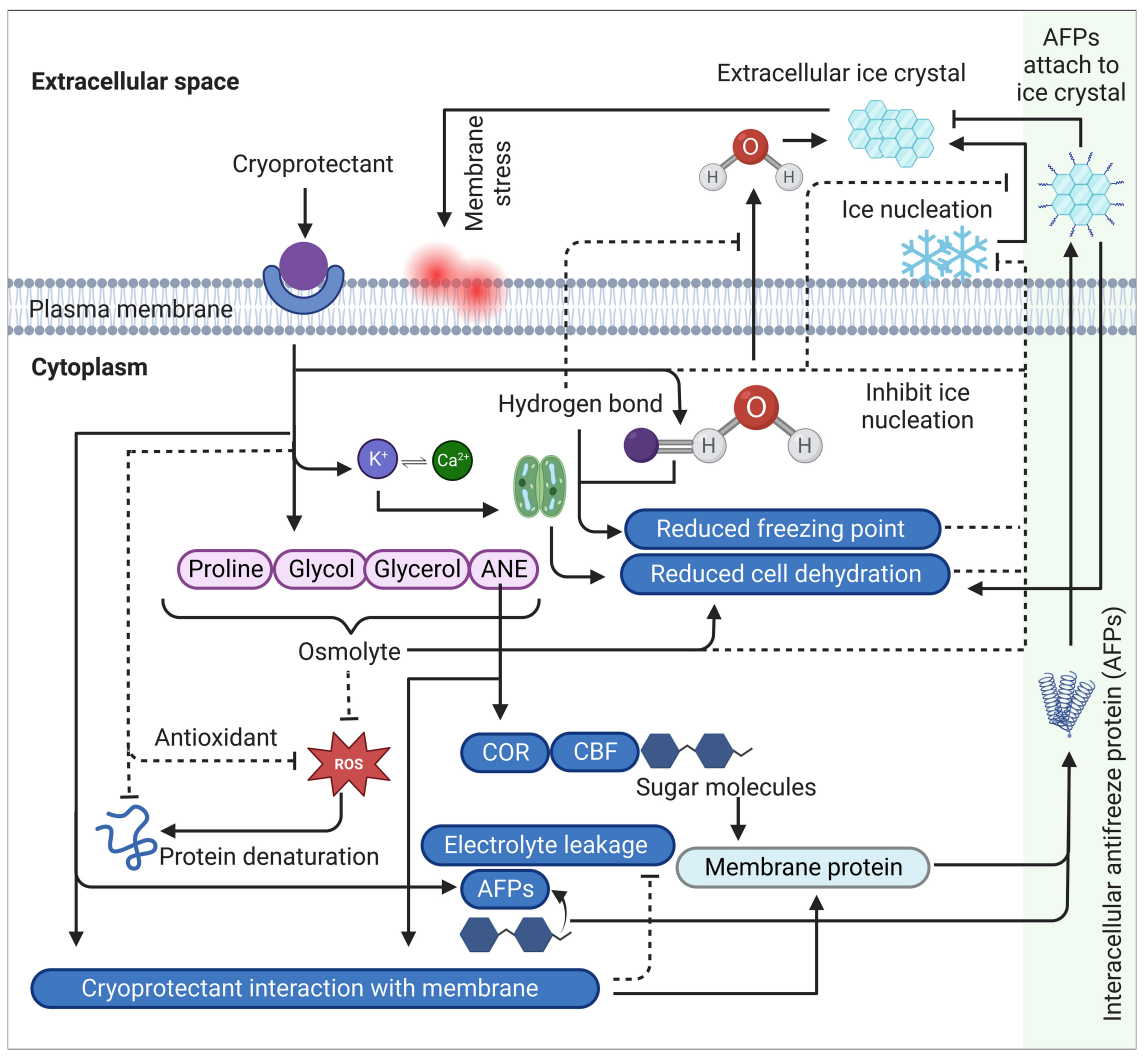
A comprehensive schematic summary of cryoprotectants mechanism of action and cold stress modulation in plants. Cryoprotectants regulate osmolytes such as proline, glycol, glycerol and ANE. The increased accumulation of these osmolytes triggers the induction of cold regulating (COR) genes through the activation of transcriptional activators called C-repeat Binding Factors (CBFs), which are involved in reducing cell dehydration, regulating membrane proteins and synthesizing antifreeze proteins (AFPs). Sugar molecules also participate in this regulation by reducing cell dehydration and modulating membrane proteins, including antifreeze proteins (AFPs). AFPs are secreted into extracellular spaces, attaching to ice crystals to prevent ice aggregation, resulting in suppressing recrystallization and reducing cell dehydration. Additionally, cryoprotectants trigger K^+^ interacting with Ca^2+^ that plays an important role in the hydrodynamic stomatal closure mechanism, leading to reduced cell dehydration. Furthermore, cryoprotectants establish hydrogen bonds with intracellular water molecules, restricting their movement into extracellular spaces and inhibiting its conversion into ice crystals. This hydrogen bonding lowers the freezing point of water. In addition, certain cryoprotectants scavenge ROS either through their antioxidant properties or through its osmoregulatory properties that contributes to maintaining cellular osmotic balance and integrity by regulating water movement across the membrane, resulting in enhanced plasma membrane stability. Collectively, these mechanisms prevent the formation of ice-nucleation sites on the membrane surface, which is a critical step in the initiation of extracellular ice crystal formation and subsequent freezing stress. Arrows indicate positive regulation while dashed line with a perpendicular line at the end indicates negative regulation. ROS, reactive oxygen species; CBF, C-repeat Binding Factors, COR, cold-responsive, AFP, antifreeze proteins, ANE, *Ascophyllum nodosum* (Seaweed) extract.

Certain cryoprotectants exhibit cryohydricity, which refers to their ability to depress the freezing point of water. This phenomenon enables plants to endure lower temperatures by modifying the colligative properties of water, including osmotic potential and increased intracellular solute concentration. Solute compounds, such as sugars, salts, and other dissolved substances, disrupt ice crystal formation and impede the progress of freezing. As a result, the freezing temperature of water within plant tissues is reduced below the standard freezing point of pure water (0°C), known as freezing point depression (Ginot et al., 2020) (**Figure 2**). The extent of this depression depends on the concentration and nature of the solutes involved, with higher solute concentrations yielding greater reductions in freezing point. This reduced freezing point enables plant cells to remain in a liquid state even at sub-zero temperatures, thus preventing or delaying ice crystal formation. At the cellular level, cryoprotectants modify water properties through hydrogen bonding interactions between water and cryoprotectant molecules. This hydrogen bonding plays a crucial role in hindering the movement of intracellular water into the extracellular space, effectively reducing ice crystal formation and minimizing cellular damage (Weng et al., 2011; Elliott et al., 2017) (**Figure 2**).

An example of an exogenous cryoprotectant capable of lowering the freezing point of plant tissues is FreezePruf, a commercially available product. FreezePruf is formulated using polyethylene glycol, potassium silicate, glycerol, silicone polyether surfactant, and a bicyclic oxazolidine anti-desiccant. These constituent molecules act as solutes or osmolytes, and their application increases solute concentration in the intracellular cytoplasmic compartments, leading to a reduction in the freezing point of these cellular regions. Francko et al. (2011) conducted a study to evaluate the effectiveness of FreezePruf on various monocot herbaceous and dicot fruit crops. Their experimental results demonstrated that the treatment with FreezePruf significantly decreased freezing-induced injury and lowered the mortality temperature by approximately 7 degrees when applied to foliage and about 1 degree when applied to open flowers. This reduction in freezing temperature is primarily attributed to the active ingredients present in FreezePruf, which act colligatively via freezing point depression as solutes. Moreover, these ingredients exert non-colligative effects by stabilizing cell membranes, thereby providing protection against freeze damage caused by ice crystal formation (Francko et al., 2011).

### 5.3 Stabilizing cellular structures

Plasma membranes are highly vulnerable to damage caused by freezing. This damage arises from several factors, including increased levels of ROS, the transition of the lipid bilayer from a fluid state to a solid-gel state, cell dehydration, and the formation of extracellular ice crystals. Collectively, these factors compromise the integrity, fluidity, and functionality of the membrane, leading to cellular damage and impaired plant survival under freeze stress. The temperature-dependent phase transition of the lipid bilayer plays a critical role in freeze-induced membrane damage. This transition reduces water diffusion across the membrane (Eze, 1991) and disrupts lipid-protein interactions, impacting membrane integrity and function (Iivonen et al., 2004). The composition of lipids, specifically the number of carbon atoms and double bonds in the lipid tail, influences the phase transition temperature. Lipids with higher unsaturation, characterized by an increased number of double bonds, exhibit a lower phase transition temperature, thereby reducing the occurrence of phase transition, leading to reduced freeze-induced membrane damage (Ambroise et al., 2020).

Cryoprotectants have been demonstrated to stabilize cellular structures and plasma membranes through various mechanisms. These mechanisms include modulating the membrane lipid phase transition, preventing protein denaturation, interacting with membrane proteins to stabilize their conformation and function, inducing membrane repair mechanisms, enhancing membrane flexibility, reducing mechanical stress, and acting as osmoregulators (Fürtauer et al., 2019). Furthermore, the osmoregulatory properties of these compounds contribute to maintaining cellular osmotic balance and integrity by regulating water movement across the membrane, resulting in enhanced plasma membrane stability (Sami et al., 2016; Arakawa et al., 2018; Fürtauer et al., 2019).

Certain commercially available cryoprotectants, such as KDL, FrostGard, and Superkelp/Vitazyme, utilize *Ascophyllum nodosum* (Seaweed) extract (ANE) as an active ingredient. ANE has been reported to alleviate low-temperature stress in plants by mitigating freezing-induced electrolyte leakage through the preservation of membrane integrity (**Figure 2**). Notably, ANE application on winter barley has been found to enhance winter hardiness and augment cold resistance (Shukla et al., 2019). Additionally, treatment with the lipophilic fraction (LPC) of ANE in Arabidopsis plants improved cold tolerance, as evidenced by the suppression of chlorosis and recovery from freezing-induced tissue damage (Rayirath et al., 2009).

Furthermore, a comprehensive analysis of the transcriptome and metabolome of LPC-treated Arabidopsis plants revealed that ANE treatment induces the expression of cold-responsive genes, including *COR15A*, *RD29A*, and *CBF* (Rayirath et al., 2009), as well as genes associated with sugar accumulation and lipid metabolism (Nair et al., 2012). Metabolite profiling of LPC-treated plants showed that the observed protection against freezing stress is mediated by the regulation of soluble sugars, sugar alcohols, organic acids, and lipophilic components such as fatty acids (Nair et al., 2012). The accumulation of sugars plays a crucial role in mitigating freezing stress by stabilizing various biological components, such as cellular membranes and membrane-bound organelles (Tarkowski and Van den Ende 2015). Interestingly, ANE treatment did not improve freezing tolerance in the *sfr4* (sensitive to freezing) mutant of Arabidopsis, which is defective in the accumulation of free sugars, suggesting that ANE-induced accumulation of soluble sugars prior to freezing stress exposure is critical for its protective effects (Nair et al., 2012).

At the molecular level, ANE-mediated cold tolerance in plants is attributed to the regulation of key transcription factors and genes encoding cryoprotective proteins. For instance, ANE-treated Arabidopsis plants showed increased cold tolerance due to the downregulation of chlorophyll degradation genes, including *AtCLH1* and *AtCLH2*, resulting in an increased chlorophyll content. ANE-mediated cryoprotectants also upregulated genes responsible for proline biosynthesis, such as *5CS1* and *P5CS2*, while downregulating *ProDH*, a gene involved in proline degradation. Moreover, ANE treatment upregulated genes responsible for polysaccharide degradation (*9SEX1* and *SEX4*) and carbohydrate biosynthesis (*GOLS2* and *GOLS3*), while downregulating genes involved in sucrose degradation. ANE treatment also induced key genes involved in freezing tolerance, including galactinol synthase 2, pyrroline 5-carboxylate synthase, and acetyl-CoA carboxylase (Zamani-Babgohari et al., 2019; Ali et al., 2021). These findings provide compelling evidence supporting the role of ANE-based cryoprotectants in enhancing cellular stabilization in plants through molecular, biochemical, and physiological mechanisms.

Another class of cryoprotectants, such as Superkelp/Vitazyme, confer cold protection in plants by increasing the levels of endogenous proline. Proline is naturally accumulated compound in plants in response to cold stress as it is water-soluble and electrically neutral at neutral pH, which helps maintaining cell osmotic potential and protecting against cold damage (Verbruggen and Hermans, 2008). The application of exogenous cryoprotectants containing proline significantly elevates the endogenous proline levels and enhances cold tolerance. Studies have shown that proline-mediated cryoprotectant treatments in rapeseed shoots increase cold tolerance and reduce electrolyte leakage, which is an indicator of membrane injury (Jonytiene et al., 2012). Additionally, proline-treated *Brassica juncea* has been shown to protect isolated thylakoid membranes from photoinhibition (Bhandari and Nayyar, 2014) and improve the maintenance of chloroplast ultrastructure and membrane integrity in tobacco plants (Van Rensburg et al., 1993). Moreover, proline-stimulating cryoprotectants exert their protective effects by interacting with plasma membrane-bound proteins and enzymes. These compounds act as hydrotropes to stabilize enzymes and biomembranes, owing to the ability of proline to form hydrophilic colloids with water and a hydrophobic backbone for protein interactions. This helps maintain the structure and conformation of proteins necessary for their functional integrity. Additionally, proline functions as a chaperone to prevent membrane proteins from denaturation (Rajendrakumar et al., 1994; Zhang et al., 2011).

### 5.4 Cryoprotectant interactions with ice crystals: inhibiting nucleation and their growth

Certain cryoprotectants possess the ability to inhibit the formation of ice nuclei, which serve as the initial points for ice crystal growth. These cryoprotectants also prevent the fusion of smaller ice crystals into larger ones, which could otherwise lead to cellular damage. For example, cryoprotectants containing antifreeze proteins (AFPs) have been shown to bind to the surface of ice crystals and inhibit their growth, thus preventing the formation of large ice crystals in the extracellular space (**Figure 2**). These molecules can also inhibit ice recrystallization, a phenomenon that can occur during repeated freeze-thaw cycles or prolonged exposure to low temperatures (Meyer et al., 1999; Wisniewski and Fuller, 1999; Griffith and Yaish, 2004). The effect of three synthetic analogues based on naturally occurring antifreeze peptides, AFPW, DCR26, DCR39, was investigated on carrot and cherry fruits (Kong et al., 2016; Kong et al., 2017). The *in vitro* experimental results showed that these compounds modified ice crystal morphology, resulting in reduced cell damage by ice crystals (Kong et al., 2017). Interestingly, certain plant AFPs share sequence homology with plant pathogenesis-related (PR) proteins including endochitinases (PR3), endo-β-1,3-glucanases (PR2), and thaumatines (PR5) (Antikainen et al., 1996; Pearce, 2001). This suggests that AFPs may serve a dual role in cold survival by mitigating freeze-related damage and providing defense against psychrophilic pathogens (e.g., ice-nucleating bacteria) (Griffith and Yaish, 2004).

The precise molecular mechanism underlying the interaction between cryoprotectants, particularly those containing AFPs, and ice crystals is not yet fully understood. Several factors that facilitate the binding of AFPs to ice nuclei have been identified. These include the amino acid composition of AFPs, with specific residues playing a role in the binding process (Davies and Sykes, 1997). Additionally, the secondary structure of these compounds, such as beta-strand-rich proteins, has been implicated in their ice-binding ability (Lu et al., 2002). Motifs such as the presence of N-acetyl groups at C-2 peptide chains with O-glycosidic linkages and gamma-methyl groups at threonine have also been identified as potential contributors to the ice-binding mechanism (Urbańczyk et al., 2017; Chakraborty and Jana, 2018). These ice-binding sites are primarily hydrophobic, although recent molecular studies have shown that both hydrophobic and hydrophilic properties contribute to the ice-binding mechanism (Hudait et al., 2018). Furthermore, AFPs and AFP-mediated cryoprotectants are known to form cages around the methyl groups on the ice-binding surface, organizing surrounding water molecules into an ice-like lattice and creating a quasi-liquid-like layer between water and the already formed ice crystals (Satyakam et al., 2022). However, despite these insights, the precise molecular mechanism by which certain cryoprotectants interact with ice crystals remains to be fully elucidated, and further research is needed to better understand this intriguing phenomenon.

In some cases, exogenously applied cryoprotectants can inhibit ice-nucleating bacteria by using inorganic salts or organic polymers to create a vapor barrier on the leaf surface, offering modest frost and freeze protection (Francko et al., 2011). An example of such cryoprotectants is a successful patent by Glenn et al. (2001), which describes a slurry comprising particulate materials that prevent ice crystal formation on the leaf surface at freezing temperatures. Ice-nucleating active (INA) bacteria, such as *Pseudomonas syringae* van Hall and *Erwinia herbicola* (Lihnis) Dye, possess a unique capability to catalyze ice formation at temperatures slightly below the freezing point (approximately -2°C) (Lindow et al., 1982). The ice-nucleation capability of INA bacteria is attributed to their specialized hydrophilic-hydrophobic ice-active sites (Pandey et al., 2016). In natural conditions, frost damage to plants typically occurs between -2°C and -5°C. Within this temperature range, ice crystals form from supercooled water within the plants, propagating throughout the intercellular and intracellular spaces and causing damage. In the absence of ice nucleation sites, water in plant tissues can remain in a supercooled state without freezing until the temperature becomes low enough for the most active ice nucleation site associated with the plant to catalyze the crystallization of supercooled water (Lindow et al., 1982). INA bacteria, found ubiquitously on plant surfaces, prevent supercooling by initiating the ice nucleation process using specialized ice-nucleating proteins (INPs) attached to their outer cellular membrane (Pandey et al., 2016), ultimately leading to freeze injury alleviation (Roeters et al., 2021; Lukas et al., 2022).

### 5.5 Reducing cell dehydration

At sub-zero temperatures, freeze-induced dehydration stress triggers changes in the lipid bilayer configuration of cell membranes, leading to a transition from a lamellar phase to a hexagonal II phase. The formation of hexagonal II phase membranes is facilitated by decreased water content, an increased number of intracellular membranes, and the presence of specific lipids such as phosphatidylethanolamine and sterols in the bilipid membrane (Uemura et al., 1995). Lipid unsaturation in plant cells plays a pivotal role in mitigating freeze damage and enhancing cold resistance. This is largely due to the properties of unsaturated fatty acids within the phospholipids of the cell membrane. These fatty acids have double bonds that introduce kinks in the fatty acid chains, preventing them from packing tightly and solidifying at low temperatures. Consequently, a higher degree of lipid unsaturation typically decreases freeze damage in plants. This is an adaptive strategy that many cold-tolerant plant species employ, synthesizing and accumulating unsaturated fatty acids under low-temperature exposure.

Alongside the structural changes in the lipid bilayer, dehydration stress can also induce expansion-induced lysis, where negative osmotic pressure drives water movement from the cell interior towards the extracellular space, resulting in a reduction in cell volume. This phenomenon causes the plasma membrane to invaginate and form endocytic vesicles, leading to a loss of plasma membrane surface area. Upon rewarming, the melted extracellular water reenters the cell, causing the cell to burst before regaining its original volume. In contrast, in cold-acclimated cell protoplasts, the volume reduction triggers the formation of exocytotic extrusions that do not decrease the membrane surface area and do not cause cell bursting upon rewarming (Uemura et al., 1995; Ambroise et al., 2020).

The application of specific exogenous cryoprotectants, such as Anti-Stress 2000 (Terra Tech, Eugene, OR) and Wilt-Pruf (Wilt-Pruf Products, Essex, CT), has been demonstrated to mitigate dehydration risk in plants during freezing and thawing cycles. This protective effect is attributed to the anti-desiccant properties of these compounds, which reduce water loss from plant tissues resulting from osmotic stress induced by ice crystal formation (**Figure 2**). By preserving cellular integrity and preventing dehydration-induced damage, these cryoprotectants play a vital role in plant protection. As discussed earlier, certain cryoprotectants contain molecules capable of lowering the freezing points of plant tissues. Additionally, these compounds contribute significantly to the prevention of cellular dehydration. For instance, potassium, a key component of several cryoprotectants, plays a crucial role in the hydrodynamic stomatal closure mechanism and helps maintain cellular osmotic balance (**Figure 2**). This regulatory mechanism of potassium is essential for preserving cellular integrity during freezing and thawing cycles (Cakmak, 2005; Wang et al., 2013).

### 5.6 Scavenging reactive oxygen species

Reactive Oxygen Species (ROS) are partially reduced or excited derivatives of molecular oxygen (O_2_), which naturally arise as a normal byproduct of aerobic life (Mittler, 2017; Sies and Jones, 2020). In contrast to O_2_, ROS are highly reactive and autonomously produced in various cellular compartments, leading to the oxidation of lipids, proteins, DNA, RNA, and many small cellular molecules. The high reactivity of ROS is attributed to their altered chemical composition, enabling them to engage in electron donation or transfer an excited energy states to accepter molecules (Mittler et al., 2022). The major forms of ROS in cells include free radicals like superoxide anion radical (O_2_•−) and hydroxyl radical (OH•), and non-radicals like hydrogen peroxide (H_2_O_2_) and singlet oxygen (^1^O_2_), and various forms of organic and inorganic peroxides (Mittler, 2017; Sies and Jones, 2020). ROS are primarily formed in chloroplast, mitochondria and peroxisomes. There are secondary sites including endoplasmic reticulum, cell membrane, cell wall and the apoplast, typically equipped with molecules having a high redox potential to reduced or excite molecular oxygen (Das and Roychoudhury, 2014; Mittler, 2017). ROS can be produced both passively by housekeeping enzyme or as by-products of metabolic pathways such as photosynthesis and respiration, and actively by specialized oxidase enzymes such as the respiratory burst oxidase homologues (RBOHs) proteins, which are the functional equivalent of mammalian NADPH oxidase (NOX proteins) (Mittler et al., 2022). Superoxide radical is the first by-product produced at the apoplast through the function of RBOHs proteins, which is subsequently dismutated by the action of superoxide dismutase (SOD) to H_2_O_2_. The membrane permeable H_2_O_2_ is the premier signaling molecule, playing an important role in regulating various cellular metabolic activities associated with growth, development, cell expansion and response to environmental stimuli (Baxter et al., 2014; Smirnoff and Arnaud, 2019). Hydrogen peroxide interacts with ferrous ions (Fe^2+^), forming OH•, the highly reactive form of ROS, through Fenton reaction (Mittler, 2017).

ROS display a dual role in plant cells, depending on their accumulation levels. A moderate, “steady-state” level of ROS is required for the progression of various fundamental biological processes, including cellular proliferation, differentiation and important signaling reactions in cells, but are also the unavoidable toxic byproducts of aerobic metabolism. As signaling molecules, ROS enables cells to rapidly respond to environmental stimuli and triggers plant’s cellular defense signaling cascade (Chi et al., 2013). Under optimal growth conditions, plants maintain a basal level of ROS serving as signaling molecules to sense elevated atmospheric oxygen levels and to monitor different metabolic reactions. However, different biotic and abiotic stress (i.e., freezing stress) disrupt the cellular homeostasis, uncouple metabolic pathways and leads to an increased production of ROS (Suzuki and Mittler, 2006; Miller et al., 2010). The elevated accumulation of ROS in cells leads to toxic oxidative stress, which adversely damages cellular components including protein denaturation, membrane lipids degradation through peroxidation (Heidarvand and Maali-Amiri, 2013) and oxidation and damaging of DNA, RNA, protein and membrane (Mittler, 2002; Mittler, 2017; Sharma et al., 2021), leading to cell death. The ROS-induced cell death is mediated through a programmed physiological and genetic pathway such as ferroptosis or regulated necrosis (Mittler, 2017). Plants pose ROS-scavenging mechanisms to detoxify the excessive ROS and maintain the balance between scavenging and production. This include the various antioxidative enzymes such as superoxide dismutase (SOD), ascorbate peroxidase (APX), catalase (CAT), glutathione peroxidase (GPX), peroxiredoxin (PRX), monodehydroascorbate reductase (MDHAR), and dehydroascorbate reductase (DHAR), and non-enzymatic antioxidants including ascorbic acid (AsA), reduced glutathione (GSH), α-tocopherol, carotenoids, flavonoids, and the osmolyte proline. The presence of these antioxidative systems in plants helps maintain a basal non-toxic level of ROS in cells, which serve as signaling molecules and promotes normal cellular processes (Miller et al., 2010; Das and Roychoudhury, 2014).

The application of certain cryoprotectants, such as Basfoliar® Frost Protect or COMPO® Frost Protect, has been shown to effectively mitigate ROS toxicity in plant cells. These cryoprotectants contain α-tocopherol, a lipophilic antioxidant that exhibit ROS and lipid radicals scavenging properties (Gill and Tuteja, 2010; Das and Roychoudhury, 2014) (**Figure 2**). Among the four isoforms of tocopherol (α-, β-, γ-, δ-), α-tocopherol has the highest antioxidant capability (Das and Roychoudhury, 2014). The antioxidant activity of tocopherol is primarily attributed to its ability of donating phenolic hydrogens to lipid free-radicals (Kamal-Eldin and Appelqvist, 1996). These compounds are known for their ability to inhibit lipid peroxidation, preserve the integrity and fluidity of photosynthesizing membranes and prevent membrane damage by interacting with O_2_ molecule and quenching reactive oxidative anions in which the reactivity of the highly reactive ROS, such as ^1^O_2_, is substantially reduced. It has been estimated that a single molecule of α-tocopherol can neutralize up to 120 molecules of ^1^O_2_ through resonance energy transfer (Gill and Tuteja, 2010; Das and Roychoudhury, 2014; Sadiq et al., 2019). Multiple studies demonstrated that increased accumulation of α-tocopherol in winter wheat leaves was associated with enhanced frost tolerance (Janeczko et al., 2018). Additionally, the application of exogenous α-tocopherol cryoprotectants has been found to enhance abiotic stress tolerance in onions by reducing the level of endogenous H_2_O_2_ and lipid peroxidation, and increasing the activity of antioxidative enzymes (i.e., SAD, CAT, APX, and GPX) and non-enzymatic antioxidants (i.e., AsA and GSH) (Semida et al., 2016).

Additionally, cryoprotectants, such as Frost Shield^®^ (Maz-Zee S.A. International) and *CROPAID* ^®^ *NPA* ^®^ (Natural Plant Antifreeze, Bimas, Turkey), can enhance plant tolerance to excessive ROS levels through sequestering or replacing cellular free iron with other minerals. These cryoprotectants contain minerals and amino acids in their composition. Minerals, such as Mn^2+^ that is found in complex with amino acid, peptides, nucleotides and carbohydrates, can replace iron to prevent its toxicity during oxidative stress (Mittler, 2017). The presence of free iron in the form of Fe^2+^ is considered crucial for ROS toxicity due to its role in the Fenton reaction, where it catalyzes the generation of highly reactive hydroxyl radicals. By sequestering or substituting free iron with alternative minerals (i.e., Mn^2+^), cryoprotectants can disrupt the Fenton reaction and attenuate the production of damaging hydroxyl radicals. In addition, these manganese complexes are shown to effectively scavenge O_2_ **^•^** ^−^, H_2_O_2_ and OH**^•^** (Slade and Radman, 2011; Mittler, 2017). Overall, cryoprotectants modulate the equilibrium between ROS production through NADPH and ROS scavenging through various enzymatic and non-enzymatic antioxidants. However, it should be acknowledged that the specific molecular mechanisms can vary based on the type of cryoprotectant, plant species, and cellular conditions. Further research is required to gain a comprehensive understanding of these mechanisms.

## 6 Conclusion

Plants utilize advanced molecular mechanisms to perceive and respond to cold stress. Transmembrane proteins function as initial sensors for cold stress signals, triggering a cascade of molecular events that involve the generation of secondary messengers such as calcium ions (Ca^2+^), reactive oxygen species (ROS), and inositol phosphates. Among these, calcium signaling plays a critical role, activating downstream phosphorylation cascades and inducing the transcription of cold-responsive genes, including the cold-regulated (COR) genes. Cold stress imposes significant challenges on plant tissues, leading to membrane rigidification and dehydration caused by freeze-induced damage. In an effort to mitigate these adverse effects, plants have evolved strategies encompassing both avoidance and tolerance mechanisms. Tolerance mechanisms involve the gradual acclimatization of plants to decreasing temperatures, allowing them to incrementally accumulate cold tolerance. In contrast, avoidance mechanisms are predicted on the presence of cryoprotectant molecules such as potassium ions (K^+^), proline, glycerol, and antifreeze proteins (AFPs).

Cryoprotectants operate by increasing intracellular solute concentration, thereby lowering the freezing point and obstructing ice formation. These molecules also exhibit potent antioxidant properties, efficiently scavenging ROS and preventing protein denaturation. By forming extensive hydrogen bonds with water molecules, cryoprotectants can also alter water properties, limiting intercellular water movement, and minimizing extracellular ice crystal formation (**Figure 2**). This action consequently reduces cell dehydration and mitigates freeze-induced cellular damage. In addition, cryoprotectants aid in the preservation of plasma membrane fluidity and the reduction of electrolyte leakage, effectively shielding plant cells against freeze-induced injury. The intricate relationship between these cryoprotectant molecules and cellular processes enhances the plant’s resilience to cold stress and bolsters survival in freezing environments. Taken together, the deployment of cryoprotectants represents a sophisticated adaptive strategy utilized by plants to counter the damaging effects of cold stress, thereby facilitating their acclimation and survival in freezing conditions. Nevertheless, a comprehensive understanding of these mechanisms warrants further research. It is essential to elucidate the physiological and molecular mechanisms of these compounds and evaluate their potential impact on crop productivity and sustainability. Such efforts will undoubtedly contribute to the ongoing advancement of plant biology and the broader field of environmental science.

## Author contributions

KJ conducted literature searches, prepared schematic diagrams, and wrote the first draft. AS contributed to figure preparation and literature review. SS oversaw the study, provided edits to improve clarity and readability, and ensured the overall quality of the manuscript. All authors contributed to the article and approved the submitted version.
